# Role of aspartate ammonia-lyase in *Pasteurella multocida*

**DOI:** 10.1186/s12866-020-02049-2

**Published:** 2020-12-03

**Authors:** Zui Wang, Li Li, Peng Liu, Chen Wang, Qin Lu, Lina Liu, Xiaozhong Wang, Qingping Luo, Huabin Shao

**Affiliations:** 1 Institute of Animal Husbandry and Veterinary Sciences, Hubei Academy of Agricultural Sciences, Special one, Nanhuyaoyuan, Hongshan District, Wuhan, 430064 China; 2 Animal Disease Prevention and Control Center of Yichang, Yichang, 443000 China; 3 Key Laboratory of Prevention and Control Agents for Animal Bacteriosis, Special 1, Nanhuyaoyuan, Hongshan District, Wuhan, 430064 China

**Keywords:** *Pasteurella multocida*, Aspartate ammonia-lyase, Iron acquisition, Virulence

## Abstract

**Background:**

*Pasteurella multocida* is responsible for a highly infectious and contagious disease in birds, leading to heavy economic losses in the chicken industry. However, the pathogenesis of this disease is poorly understood. We recently identified an aspartate ammonia-lyase (*aspA*) in *P. multocida* that was significantly upregulated under iron-restricted conditions, the protein of which could effectively protect chicken flocks against *P. multocida*. However, the functions of this gene remain unclear. In the present study, we constructed *aspA* mutant strain △*aspA*::*kan* and complementary strain C△*aspA*::*kan* to investigate the function of *aspA* in detail.

**Result:**

Deletion of the *aspA* gene in *P. multocida* resulted in a significant reduction in bacterial growth in LB (Luria-Bertani) and MH (Mueller-Hinton) media, which was rescued by supplementation with 20 mM fumarate. The mutant strain △*aspA*::*kan* showed significantly growth defects in anaerobic conditions and acid medium, compared with the wild-type strain. Moreover, growth of △*aspA*::*kan* was more seriously impaired than that of the wild-type strain under iron-restricted conditions, and this growth recovered after supplementation with iron ions. *AspA* transcription was negatively regulated by iron conditions, as demonstrated by quantitative reverse transcription-polymerase chain reaction. Although competitive index assay showed the wild-type strain outcompetes the *aspA* mutant strain and △*aspA*::*kan* was significantly more efficient at producing biofilms than the wild-type strain, there was no significant difference in virulence between the mutant and the wild-type strains.

**Conclusion:**

These results demonstrate that *aspA* is required for bacterial growth in complex medium, and under anaerobic, acid, and iron-limited conditions.

## Background

*Pasteurella multocida* is a capsulated, Gram-negative facultative anaerobic bacterium responsible for fowl cholera in poultry, leading to great economic losses in commercial layer flocks and local chicken breeds [1]. *P. multocida* is currently classified into five serogroups (A, B, D, E, and F) based on its capsular antigens, and fowl cholera is mainly caused by strains of serovars A, F, and very rarely D [2]. Once a chicken flock becomes infected with the bacterium, it may become endemic and difficult to remove, resulting in repeated infectious episodes [3]. However, the molecular basis of *P. multocida* pathogenesis is still poorly understood.

Aspartate ammonia-lyase (*aspA*) has been identified in various other Gram-negative bacteria, including *Salmonella enterica* [4], *Actinobacillus pleuropneumoniae* [5] and *Escherichia coli* [6]. This enzyme is involved in catalyzing the reversible conversion of L-aspartate to form fumarate and release ammonia [7], and plays a vital role in the production of L-aspartate [8]. The addition of L-aspartate significantly increased the survival of wild-type, but not *aspA* mutant, *Y. pseudotuberculosis* in minimum essential medium at pH 4.5 [9]. In addition to its role in an acid-survival system, *aspA* is also important for anaerobic respiration, and fumarate produced by the decomposition of L-aspartate can serve as a terminal electron acceptor under anaerobic conditions [10]. The *aspA* gene was significantly upregulated under iron-restricted conditions in various bacteria including *C. jejuni* [11], *A. pleuropneumoniae* [12], *Edwardsiella ictaluri* [13], and *P. multocida* [14], suggesting that *aspA* might be related to iron acquisition. Meanwhile, an iron-restricted environment often triggers the expression of virulence factors in pathogens [15], indicating the need to determine if *aspA* is an important virulence factor in *P. multocida*.

In the present study, we constructed *aspA* mutant strain △*aspA*::*kan* and complementary strain C△*aspA*::*kan* to investigate the role of *aspA* in the growth of *P. multocida* in complex medium, and under anaerobic, acid, and iron-limited conditions, and in *P. multocida* infection in vivo.

## Results

### Identification of *P. multocida* △*aspA*::*kan* mutant

**Fig. 1:**
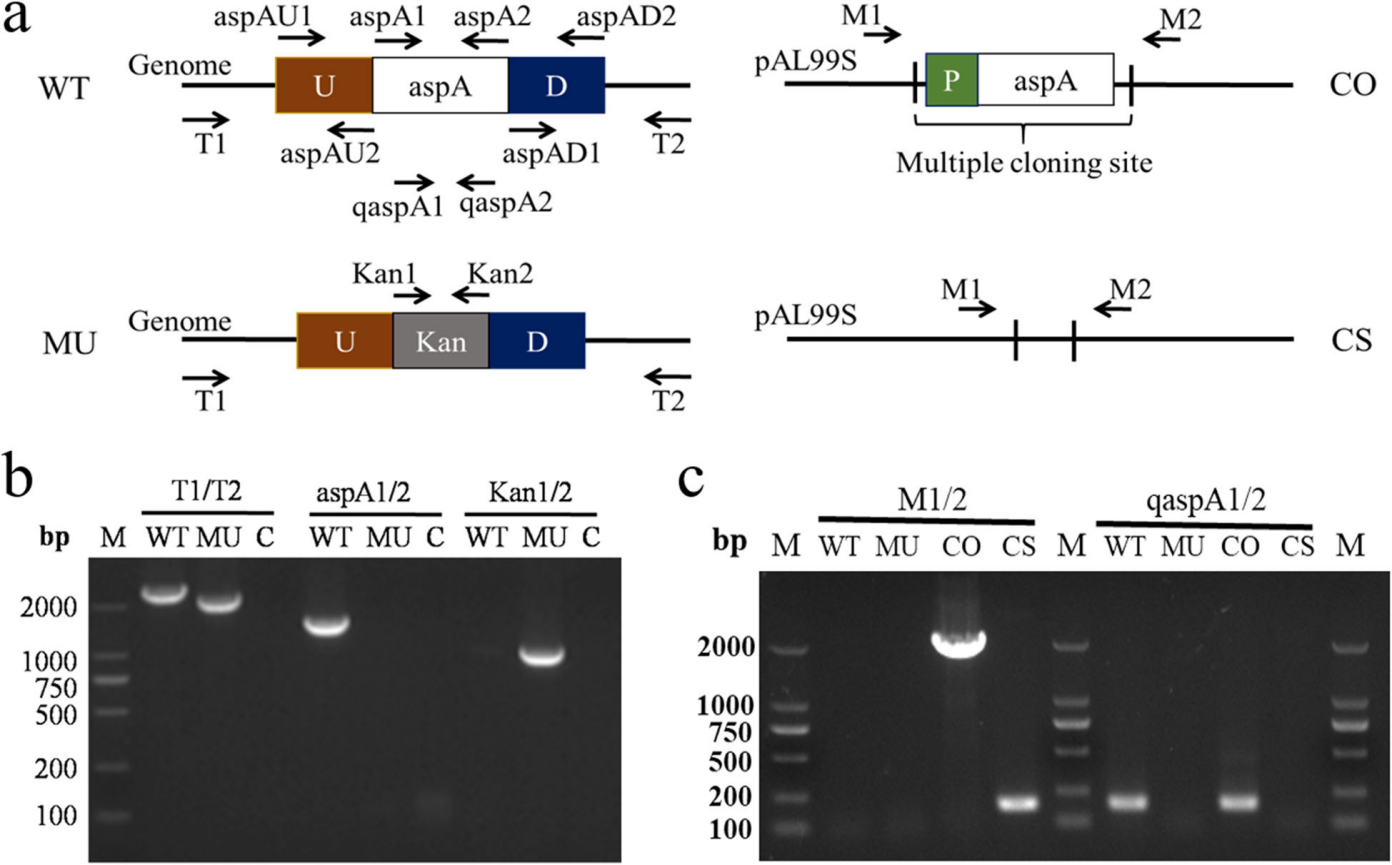
Construction of *aspA* mutant of *P. multocida* C48–1. **a** Schematic showing deletion of the target *aspA* gene. **b** Agarose gel electrophoresis confirmed the genotype of the constructed mutant. The wild-type (WT) and mutant strains (MU) were identified using three primer pairs: T1/2, aspA1/2, and Kan1/2 and C standed for negative control. **c** Confirmation of the complemented strain by PCR (left) and RT-PCR (right). The WT, MU, CO (complemented strains) and CS (mutant strains harboring an empty vector) were identified using two primer pairs: M1/2 (outside the multiple cloning site of vector) and qaspA1/2

The whole *aspA* gene (1419 bp) was replaced with a 902 bp kanamycin-resistance cassette using allelic exchange through a recombinant suicide vector. The mutant strain △*aspA*::*kan* was confirmed by PCR screening using primers T1/2, aspA1/2, and Kan1/2 (Fig. 1a and Table 2). Primers T1/2 were both designed outside the homology arms of *aspA*. The amplicon size of the deleted alleles was 517 bp less than the wild-type. Primers aspA1/2 were designed to target the whole *aspA* gene. The amplification product was present in the parent strain but not in the *aspA* mutant. In addition, primers Kan1/2 were designed to target the whole Kan cassette, which was only present in the *aspA* mutant (Fig. 1b). To further characterize the *aspA* mutant, the expressions of *aspA* were detected in the parent strain C48–1 and complementary strain C△*aspA*::*kan* but not in the mutant strain △*aspA*::*kan* or control strain △*aspA*::*kan* (pAL99S) (Fig. 1c), indicating that the *aspA* gene was successfully deleted from C48–1.

### *AspA* is essential for growth of *P. multocida*

**Fig. 2:**
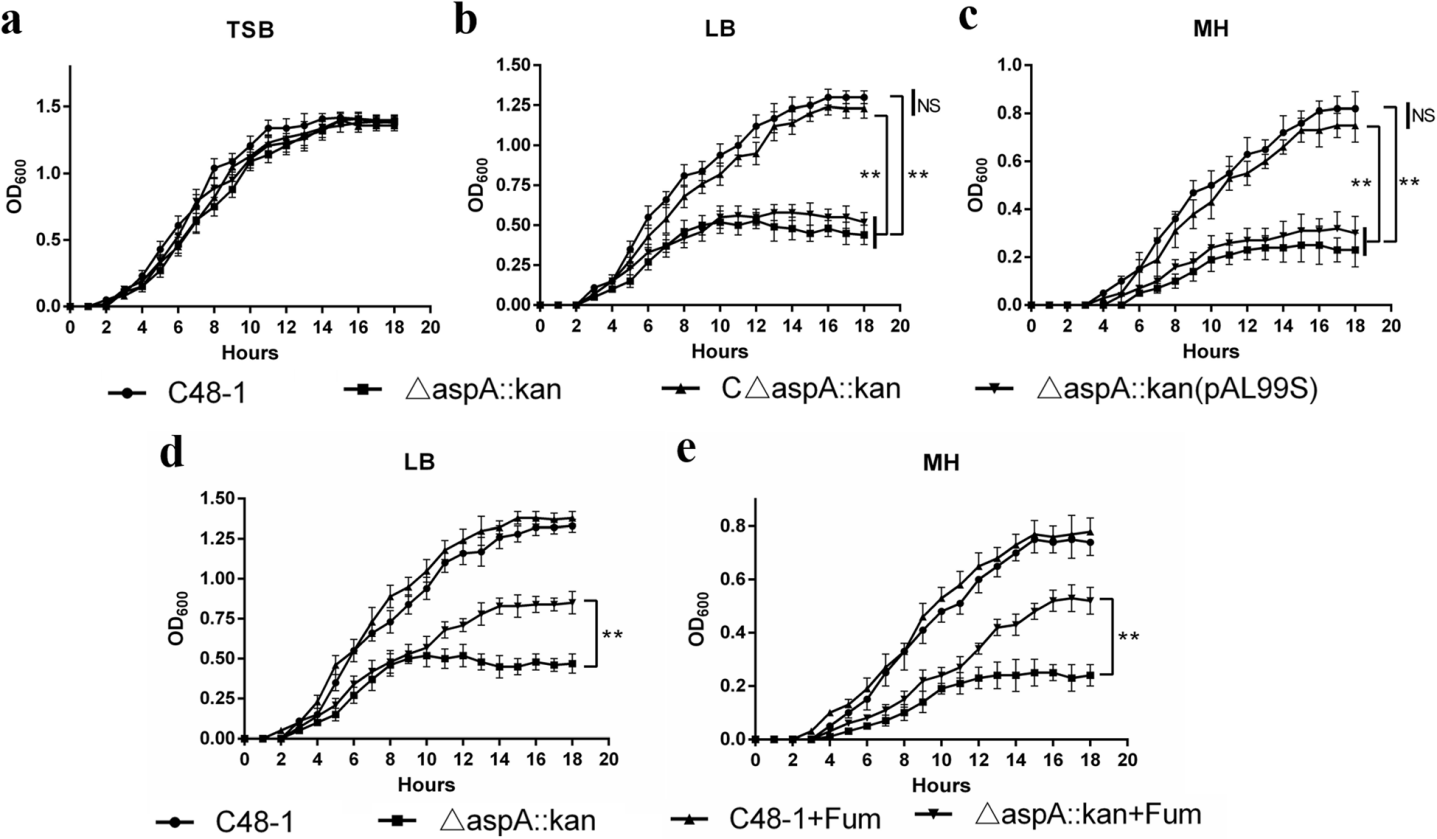
Growth of C48–1, △*aspA*::*kan*, C△*aspA*::*kan* and △*aspA*::*kan* (pAL99S) in complex media under aerobic conditions. The growth curves were measured in (**a**) TSB, (**b**) LB and (**c**) MH. Growth of C48–1 and △*aspA*::*kan* in LB (**d**) and MH (**e**) supplemented with fumarate. The growth curves of C48–1 and △*aspA*::*kan* were determined after incubation in LB and MH with or without 20 mM fumarate at 37 °Cfor 18 h. Data analyzed by Student’s *t*-test. * *P* < 0.05; ** *P* < 0.01 and NS (non-significant) for *P*>0.05

We compared the growth rates of C48–1, △*aspA*::*kan*, C△*aspA*::*kan* and △*aspA*::*kan* (pAL99S) by measuring their growth curves in TSB, MH, and LB media under aerobic conditions. The four strains showed similar growth in TSB medium, but △*aspA*::*kan* and △*aspA*::*kan* (pAL99S) growth were relatively inhibited in LB and MH medium while the growth of complementation strain C△*aspA*::*kan* was restored (Fig. 2a, b, c). Loss of *aspA* delayed the time to entry into logarithmic phase and significantly reduced the maximum growth. The final OD_600_ values of the *aspA* mutant were 0.44 in LB (*P* < 0.01) and 0.23 in MH (*P* < 0.01), compared with 1.30 and 0.82, respectively, for the wild-type strain. Interestingly, the growth defects of the mutant were partly recovered by supplementation of LB and MH with 20 mM fumarate prior to inoculation (Fig. 2d, e). The final OD_600_ values increased from 0.45 to 0.85 in LB (*P* < 0.01) and from 0.24 to 0.52 in MH (*P* < 0.01). These results indicated that fumarate, as the product of amino acid catabolism through *aspA*, was important for the growth of *P. multocida*.

### *AspA* is required for *P. multocida* growth under anaerobic conditions

**Fig. 3:**
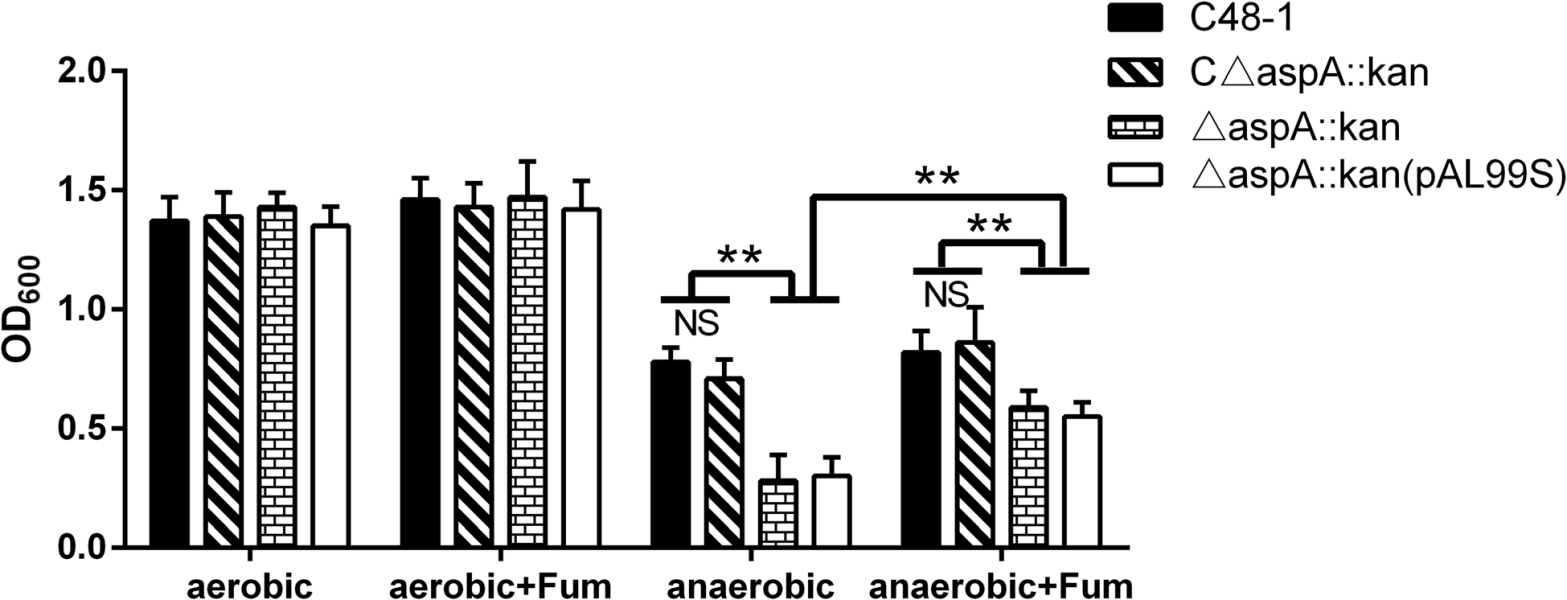
Anaerobic growth of C48–1, △*aspA*::*kan*, C△*aspA*::*kan* and △*aspA*::*kan* (pAL99S). The final OD_600_ values were determined after incubation in TSB under aerobic or anaerobic conditions with or without 20 mM fumarate at 37 °C for 16 h. Data analyzed by Student’s *t*-test. * *P* < 0.05; ** *P* < 0.01 and NS for *P*>0.05

To determine if *aspA* affected the growth of *P. multocida* under anaerobic conditions, we compared the abilities of the parent, mutant, complementary and control strains to grow under anaerobic conditions in TSB. Growth of all four strains was inhibited under anaerobic conditions, but growth of the mutant and control strains was slower than that of the wild-type and complementary strain (Fig. 3). The final OD_600_ of △*aspA*::*kan* was about 0.28, compared with 0.78 for the wild-type strain (*P* < 0.01). As expected, the growth defects of the mutant were partly recovered by supplementation with 20 mM fumarate prior to inoculation (Fig. 3). The final OD_600_ values increased from 0.28 to 0.59 under anaerobic conditions (*P* < 0.01). These results suggest that loss of *aspA* could lead to growth defects under anaerobic conditions and fumarate might play a vital role for *P. multocida* growth under anaerobic conditions.

### *AspA* is related to acid survival in *P. multocida*

**Fig. 4:**
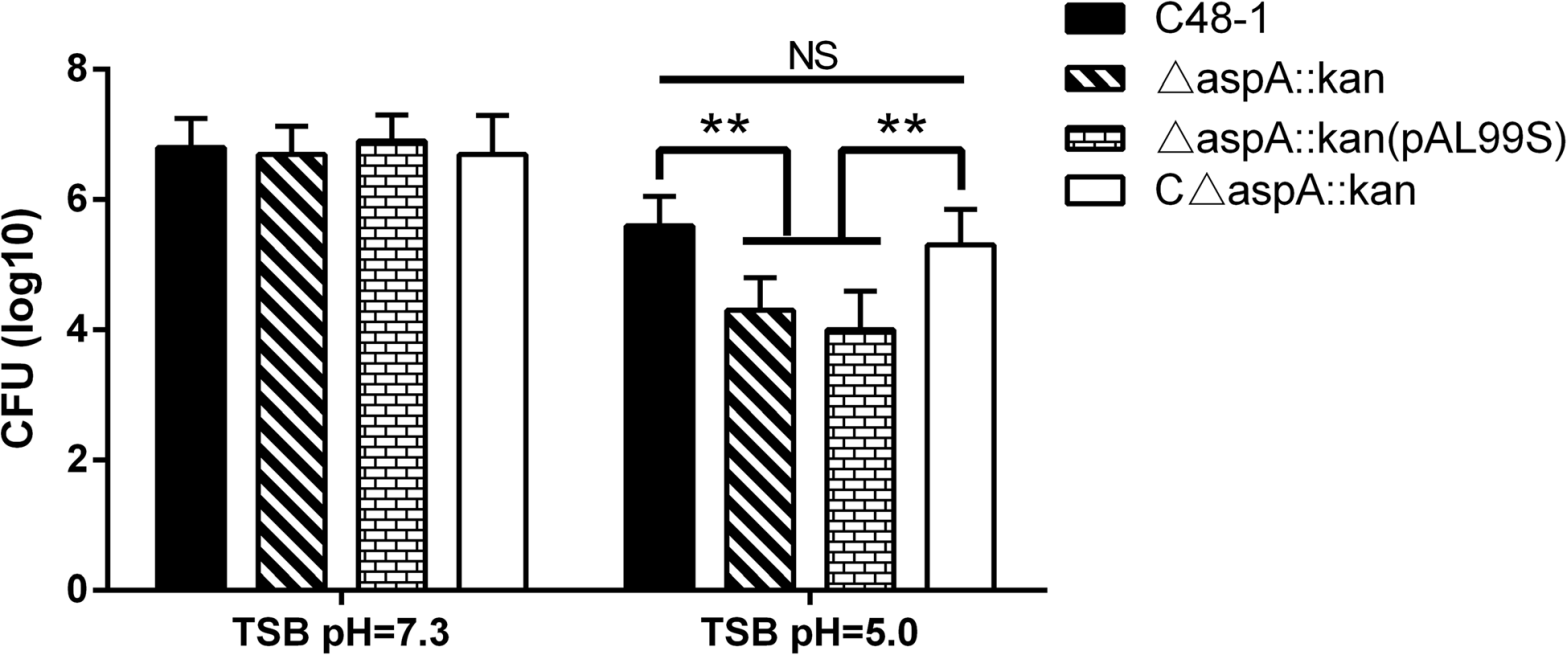
Acid growth of C48–1, △*aspA*::*kan*, C△*aspA*::*kan* and △*aspA*::*kan* (pAL99S). Overnight cultures were diluted to OD_600_ = 1. Diluted cultures were then subcultured in TSB, pH = 7.3 and TSB, pH = 5.0 at 37 °C for 1 h under aerobic conditions and the CFU/mL were counted. Data analyzed by Student’s *t*-test. * *P* < 0.05; ** *P* < 0.01 and NS for *P*>0.05

To determine if *aspA* is involved in the acid survival of *P. multocida*, we compared the abilities of the parent, mutant, complementary and control strains to grow under acid conditions in TSB in presence of oxygen. After 1 h of incubation, the densities of four strains in TSB (pH = 7.3) were approximately 6.83 × 10^6^ CFU/mL (Fig. 4). However, the density of C48–1 colonies was 3.92 × 10^5^ CFU/mL in acid medium (TSB, pH = 5), which was 12.58-fold higher than △*aspA*::*kan* (*P* < 0.01). At the same time, the density of C△*aspA*::*kan* colonies was almost the same as C48–1. This indicated that loss of *aspA* reduced the acid resistance of *P. multocida*.

### *AspA* is related to iron acquisition in *P. multocida*

**Fig. 5:**
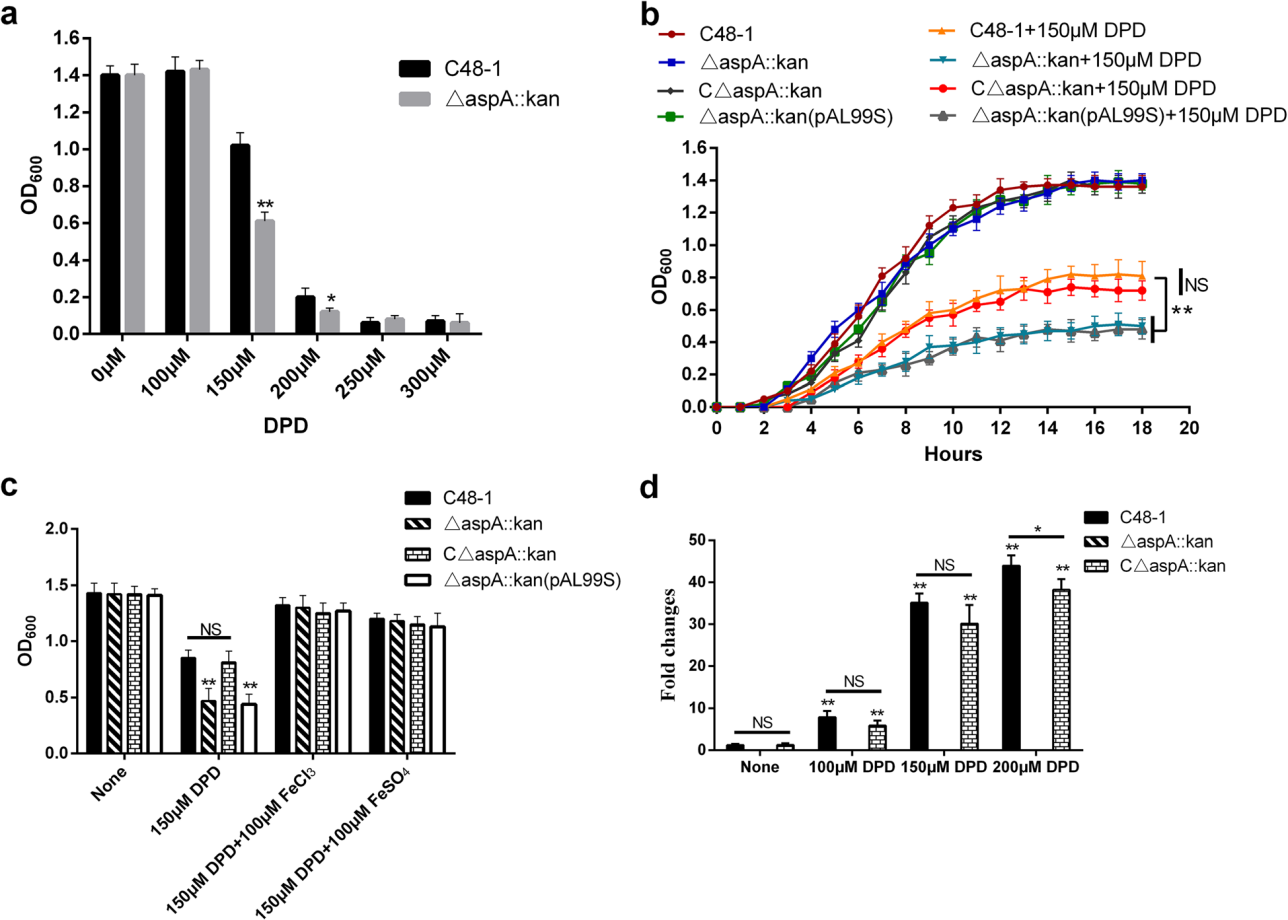
*AspA* is required for iron acquisition. **a** Effect of DPD supplementation on C48–1 and △*aspA*::*kan* growth under aerobic conditions. The final OD_600_ values were determined after incubation in TSB in different iron-limited conditions at 16 h. **b** The growth curves of C48–1, △*aspA*::*kan*, C△*aspA*::*kan* and △*aspA*::*kan* (pAL99S) were measured at 37° in TSB containing 150 μM DPD under aerobic conditions. **c** Utilization of iron sources by C48–1 and △*aspA*::*kan*, C△*aspA*::*kan* and △*aspA*::*kan* (pAL99S). Wild-type and *aspA* mutant strains were inoculated in TSB containing 150 μM DPD supplemented with FeCl_3_ or FeSO_4_ under aerobic conditions. The final OD_600_ values were determined after 16 h. **d** Transcription levels of the *aspA* gene in C48–1, △*aspA*::*kan* and C△*aspA*::*kan* in response to different iron-limited conditions. Data analyzed by Student’s *t*-test. * *P* < 0.05; ** *P* < 0.01 and NS for *P*>0.05

We examined the role of *aspA* in iron acquisition in *P. multocida*. The effect of iron depletion was determined by measuring the end-point OD of cultures in TSB containing the iron chelator 2,2′-dipyridyl (DPD) at concentrations of 0–300 μM under aerobic conditions. The DPD sensitivities of the wild-type and mutant strains were compared (Fig. 5a). The growth of both strains was inhibited in TSB supplemented with 150 and 200 μM DPD, and growth was almost absent at 250 and 300 μM DPD. The growth of the mutant was significantly slower than that of the wild-type strain in the presence of 150 μM DPD, with a reduction in OD_600_ from 1.02 to 0.6 (*P* < 0.01). We therefore chose 150 μM DPD as the optimal working concentration to create a growth curve. Loss of *aspA* delayed the time of entry into the logarithmic phase and significantly reduced the maximum growth (*P* < 0.01, Fig. 5b) while the growth of complementation strain C△*aspA*::*kan* was restored. These results suggested that the mutant strain was more sensitive to the iron-depleted environment, and that *aspA* was involved in iron acquisition in *P. multocida*. In addition, we also determined the ability of the mutant strain to utilize different iron sources in iron-depleted medium. Growth of △*aspA*::*kan* and wild-type C48–1 was inhibited in TSB containing 150 μM DPD, but growth of both was restored by addition of 100 mM FeCl_3_ or FeSO_4_, with no significant difference between the mutant and wild-type strains (Fig. 5c). We therefore concluded that loss of *aspA* affected the absorption of chelated iron rather than free iron ions. To clarify if *aspA* was negatively regulated by iron ions, we determined the relative mRNA expression levels of *aspA* in C48–1, △*aspA*::*kan*, C△*aspA*::*kan* under various iron-limited conditions. The mRNA expressions of *aspA* were significantly upregulated under iron-limited conditions in C48–1 and C△*aspA*::*kan* (*P* < 0.01, Fig. 5d). *aspA* expression increased with increasing iron chelator. These results demonstrated that *aspA* was negatively regulated by the iron concentration in *P. multocida*, indicating that *aspA* plays an important role in chelated iron acquisition in *P. multocida.*

### Effect of *aspA* deletion on biofilm formation

**Fig. 6:**
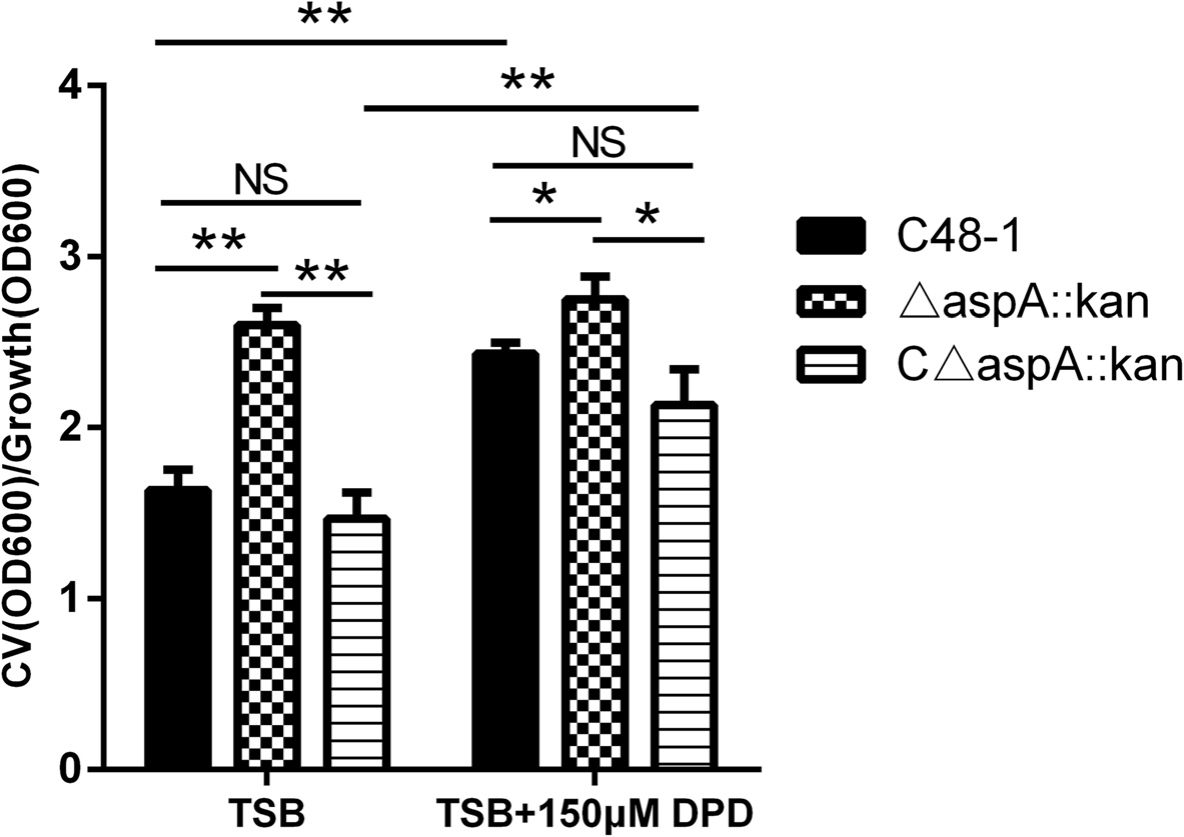
Biofilm formation assessed by crystal violet staining. Biofilms were grown in TSB with or without 150 μM DPD at 37 °C for 48 h in six-well plates under aerobic conditions. Biofilm biomass was measured by crystal violet staining and expressed relative to the final culture density. Data analyzed by Student’s *t*-test. * *P* < 0.05; ** *P* < 0.01 and NS for *P*>0.05

We explored the effect of *aspA* on biofilm formation in *P. multocida* by crystal violet staining. The mutant strain was significantly more efficient at producing biofilms than the wild-type strain C48–1 (*P* < 0.01, Fig. 6). In addition, C48–1 produced more biofilms in iron-limited than that in iron-repleted conditions (*P* < 0.01, Fig. 6), while biofilm production by the mutant was unaffected.

### Virulence of the *aspA* mutant strain

**Fig. 7:**
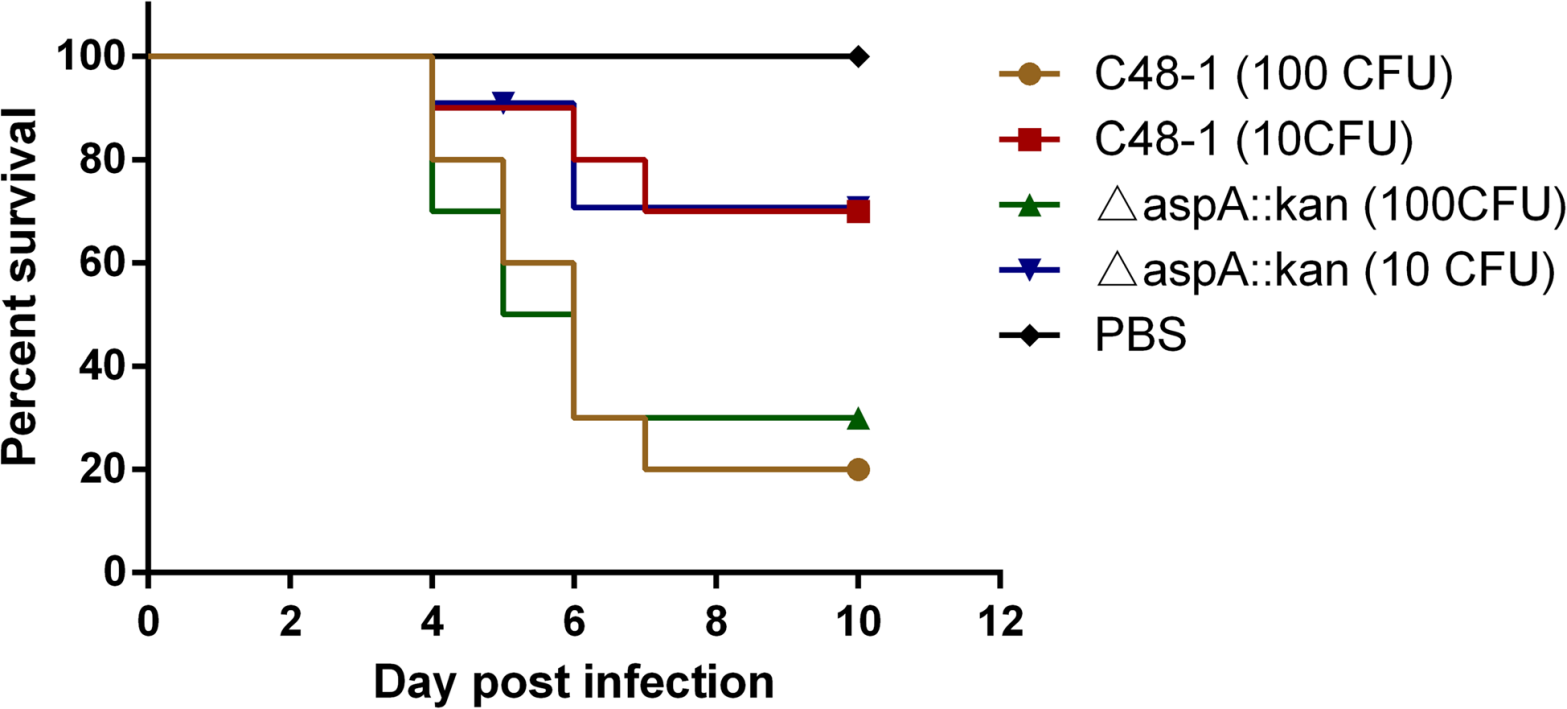
Animal challenge experiment. Healthy chickens (55 days old) were injected intramuscularly with 10 or 100 CFU of parental C48–1 or mutant △*aspA*::*kan* strain and mortality was recorded daily for 12 days after challenge

We examined the role of *aspA* in virulence in 55-day-old healthy chickens infected with the mutant and wild-type strains, respectively. At a challenge dose of 10 CFU, survival rates were the same between the mutant and wild groups (7/10, 70%) (Fig. 7). Moreover, the survival of the wild-type and mutant groups with a challenge dose of 100 CFU were 20 and 30%, respectively. These results indicated that the *aspA* gene could not be related to the virulence of *P. multocida* in chickens.

**Fig. 8:**
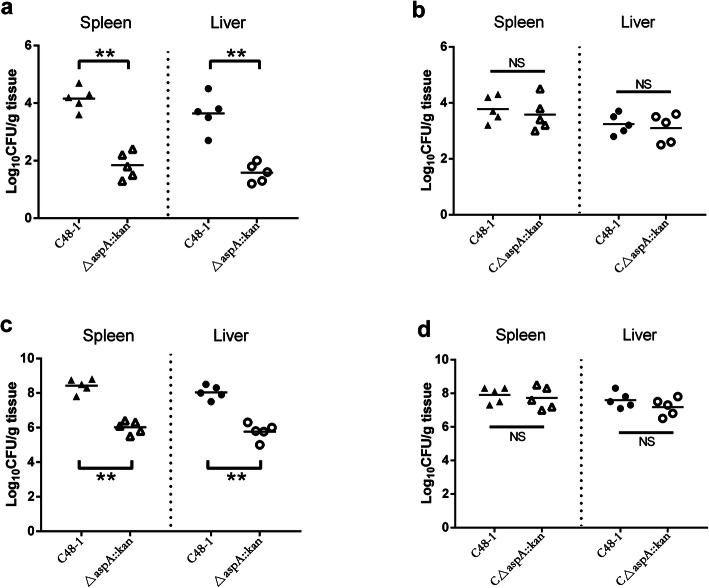
Competitive infection by the wildtype C48–1 with mutant strains △*aspA*::*kan* or complemented strains C△*aspA*::*kan* in vivo. C48–1 and △*aspA*::*kan* or C△*aspA*::*kan* were mixed at a 1:1 ratio and 200 μl of the mixture containing 100 CFU of each strain was inoculated into 55-day-old healthy chickens. Bacteria were isolated from the spleens and livers at 24 h (**a**), (**b**) and 72 h (**c**), (**d**) post-infection. Data points represent the CFU/g of individual animals in the organs indicated; bars show median values (*n* = 5). Data analyzed by Student’s *t*-test. * *P* < 0.05; ** *P* < 0.01 and NS for *P*>0.05

We further explored the role of *aspA* in the colonization dynamics of *P. multocida* during systemic infection by competitive infection of 55-day-old healthy chickens with a 1:1 ratio of C48–1 and the *aspA* mutant. The bacterial loads of the *aspA* mutant strain in the spleen (209-fold reduction, *P* < 0.01) and liver (115-fold reduction, *P* < 0.01) at 24 h post-infection were significantly reduced compared with the parent strain C48–1 (Fig. 8a). The *aspA* mutant was also significantly outcompeted by the wild-type strain in the spleen (263-fold reduction, *P* < 0.01) and liver (182-fold reduction, *P* < 0.01) at 72 h post-infection (Fig. 8c). At the same time, the colonization abilities were mostly restored in the complementary strain (Fig. 8b, d). These results showed that the wild-type strain outcompetes the *aspA* mutant strain during the infection.

## Discussion

We previously identified an aspartate ammonia-lyase (*aspA*) that was significantly upregulated under iron-restricted conditions and the protein of which could effectively protect chicken flocks against *P. multocida* [14]. This suggested that *aspA* might play an important role in the survival of these bacteria under iron-restricted conditions and could be related to the virulence of *P. multocida*. We therefore further studied the function of the *aspA* gene by constructing *aspA* mutant strain △*aspA*::*kan* and complementary strain C△*aspA*::*kan*, and assessed its functions in growth in complex medium and under anaerobic, acid, and iron-limited conditions, and during infection in vivo.

*AspA* is involved in catalyzing the reversible conversion of L-aspartate to form fumarate and release ammonia [7], which is essential for the production of L-aspartic acid as an important carbon source for various bacteria [16]. Loss of *aspA* might thus affect the utilization of aspartic acid and inhibit the growth of bacteria. In the present study, we examined the effect of *aspA* mutation on the ability of *P. multocida* to grow in different complex media. We demonstrated that △*aspA*::*kan* had dramatic growth defects in LB and MH media compared with the wild-type strain, though this was rescued by supplementation with 20 mM fumarate. Similar results were found in *C. jejuni*. The effect of the *aspA* mutation on the depletion of amino acids in MH media after 48 h growth was determined in *C. jejuni*. Only serine was significantly utilized by this mutant while proline, aspartate and glutamate couldn’t be used in comparison to the WT [17]. Fumarate, as the product of aspartate through *aspA*, could restore partially of the growth defect [17]. This suggests that amino acid catabolism involving *aspA* was crucial for the growth of *P. multocida* in complex media. In addition, fumarate was not only indispensable for bacterial growth, but also served as a terminal electron acceptor under anaerobic conditions [18]. Fumarate is produced via the malate dehydrogenase and *aspA* pathways, respectively [19]. Transcriptome analysis showed that malate dehydrogenase was downregulated under anaerobic conditions [20] whereas *aspA* was increased [21], suggesting that *aspA* produced fumarate rather than malate dehydrogenase under anaerobic conditions. In the present study, the *aspA* mutant strain △*aspA*::*kan* showed significantly decreased growth under anaerobic conditions over a 16 h incubation period compared with the wild-type strain and this growth recovered after supplementation 20 mM fumarate, suggesting that *aspA* was important for anaerobic growth in vitro. Ammonia is also a product of amino acid catabolism through *aspA*, and may be responsible for increasing the intracellular pH [9]. A novel aspartate-dependent acid-survival system involving *aspA* has been identified in *Y. pseudotuberculosis* [9]. The present study demonstrated the existence of a similar aspartate acid-survival system in *P. multocida*. The survival of C48–1 was 12.58-fold higher than that of △*aspA*::*kan* after 1 h of incubation in acid medium.

Iron is indispensable for bacterial growth and iron acquisition is an important aspect of the pathogenesis of many bacteria [22]. Various iron-uptake systems have been found to play an essential role in virulence in *P. multocida*, particularly in different animal host environments [23]. The major objective of this study was to explore the role of *aspA* in iron utilization and the pathogenesis in *P. multocida*.

Amino acid homology analysis suggested that the structural and evolutionary relationships of *aspA* were closely related to fumarase (*fumC*) [24], which is known to be related to iron acquisition in many bacteria [25–28]. As their common product, fumarate could act as an electron acceptor during iron-uptake [29]. These results suggested that *aspA* might also play a role in iron acquisition in *P. multocida*. In the present study, the mutant strain △*aspA*::*kan* was more sensitive to iron-limited conditions and showed impaired growth in TSB containing 150 μM DPD compared with the wild-type strain, thus supporting a role for *aspA* in iron-uptake. We also demonstrated that the addition of different iron ions could improve the growth of △*aspA*::*kan*, and concluded that loss of *aspA* affected the absorption of chelated rather than free iron. Iron-uptake related genes are usually modulated by iron concentration, while only a handful of genes are not [22]. We monitored the transcription of *aspA* under different iron-limited conditions and found that *aspA* was negatively regulated by iron levels in *P. multocida*. Moreover, iron is not only involved in the regulation of iron-uptake genes, but also in the formation of biofilms [30], though the promotion or inhibition of biofilm formation depends on the species of bacteria [31–34]. In this study, either iron-limited conditions or loss of *aspA* promoted the formation of biofilms by *P. multocida*. Although several iron-uptake systems are involved in biofilm formation [35, 36], the mechanism by which iron signaling regulates biofilm formation is unclear. The current results revealed that *aspA* is likely to play an important role in iron acquisition in *P. multocida.*

For most bacterial pathogens, the ability to acquire iron from the host is directly related to their virulence [37]. Knocking out iron-associated proteins can thus reduce the virulence of various bacteria, including *P. multocida* [23, 38]. Although there was no previous evidence relating *aspA* to iron acquisition, the virulence of the *aspA* mutant has been explored in other bacteria. Pigs challenged with *aspA* mutants of *A. pleuropneumoniae* showed lower lung lesion scores than those challenged with the parent controls [5]. Ability of *aspA* mutant *C. jejuni* to persist in the intestines of chickens was impaired relative to the wild-type strain [17]. However, although a competitive index assay showed the wild-type strain outcompetes the *aspA* mutant strain, there was no significant difference in the virulence of *P. multocida* between the *aspA* mutant and the wild strains in this study. The possible reasons are as follows. On the one hand, the virulence of C48–1 is too strong that deleting *aspA* is not enough to reduce the virulence. On the other hand, chickens are usually infected with *P. multocida* through the digestive and respiratory tract in the wild. An intramuscular route of infection may not reveal the role of *aspA* in virulence compared to an experiment where chickens are inoculated with *P. multocida* in a manner that reflects natural transmission such as through the mouth, nose, or conjunctiva. Moreover, *A. pleuropneumoniae* and *C. jejuni* both grow in an anaerobic environment in the host while *P. multocida* does not. The survival pressure of anaerobic environment might be higher than that of iron limiting environment. Therefore, whether *aspA* could play a vital virulence factor only in anaerobic bacteria should be further studied.

## Conclusions

In conclusion, this study demonstrated that *P. multocida aspA* was required for bacterial growth in complex medium and under anaerobic, acid, and iron-limited conditions. This study provides the first evidence for the role of *aspA* in iron acquisition. In addition, although the competitive index assay showed the wild-type strain outcompetes the *aspA* mutant strain, there was no significant difference in virulence between the mutant and the wild strains. The reasons need to be studied further.

## Materials and methods

### Bacterial strains and growth conditions

The wild-type bacterial strain used in these studies was *P. multocida* (C48–1), which was originally obtained from the China Veterinary Culture Collection Center. C48–1 is considered to be highly virulent in chickens. The bacterial strains and plasmids are described in Table 1. The *aspA* mutant △*aspA*::*kan*, was derived from the wild-type strain C48–1. Unless otherwise stated, all cultures were maintained in tryptone soy broth (TSB; Difco Laboratories, Detroit, MI, USA) in the presence or absence of 150 μM 2,2′-dipyridyl (DPD; Sigma, Santa Clara, CA, USA), and in the presence or absence of oxygen at 37 °C. The following antibiotics were added to the selection media as required: kanamycin, 100 mg/mL; chloramphenicol, 100 mg/mL; ampicillin, 100 mg/mL; spectinomycin, 100 mg/mL and gentamicin, 100 mg/mL.

**Table 1:** Strains and plasmids used in this study

Strains or plasmids	Description	Source or reference
Strains		
C48–1	*Avian P. multocida* C48–1. Capsulated and virulent	China Veterinary Culture Collection Center
△*aspA*::*kan*	*aspA* mutant strain of *Avian P. multocida* C48–1	This work
C△*aspA*::*kan*	Complemented strain of △*aspA*::*kan*	This work
△*aspA*::*kan* (pAL99S)	*aspA* mutant strain harboring an empty vector	This work
Plasmids		
pBC-Tn903	Suicide vector, Cm^R^, Kan^R^	[39]
pBC-aspA	Containing left and right arms of *aspA*, Cm^R^	This work
pBC-aspA-kan	Constructing *aspA* gene deletion mutants, Cm^R^, Kan^R^	This work
pAL99S	P. multocida expression plasmid, derivative of pAL99, Spec^R^	[40]
pAL99S-aspA	Containing the intact *aspA*, Spec^R^	This work
pET-28a	Amplifing the kanamycin resistance cassette, Kan^R^	Our Lab

*Kan*^*R*^ kanamycin resistance, *Cm*^*R*^ chloramphenicol resistance, *Spec*^*R*^ spectinomycin resistance

### Generation of the △*aspA*::*kan* mutant and complemented strains

The whole genome of *P. multocida* (C48–1) has not yet been sequenced, and all the primers were therefore based on the genomic sequence of Pm70 (GenBank accession: AE004439.1) (Table 2). *AspA* gene was deleted by allelic exchange through a recombinant suicide vector, which replaced the whole *aspA* gene with a 902 bp kanamycin-resistance cassette. Briefly, the 453 bp upstream and 447 bp downstream fragments of the *P. multocida aspA* gene were amplified using aspAU1-aspAU2 and aspAD1-aspAD2 primer sets (Table 2), respectively. The upstream and downstream fragments were fused by overlap polymerase chain reaction (PCR) using aspAU1-aspAD2 primers. The purified *aspA* deletion fragment was then cloned into pBC-Tn903 [39] at the *Kpn* I and *BamH* I restriction sites using T4 DNA ligase (Takara Bio Inc., Tokyo, Japan) to obtain the plasmid pBC-aspA. The kanamycin resistance (kan^R^) cassette amplified from pET-28a with the primers Kan-F1 and Kan-F2 (Table 2) was then inserted into the *Not* I and *Sbf* I sites of pBC-aspA to generate the plasmid pBC-aspA-*kan*. This plasmid was subsequently introduced into *P. multocida* C48–1 via electroporation to obtain a single crossover strain on TSB agar plates containing kanamycin and chloramphenicol. The second crossover strain was selected by chloramphenicol sensitivity and kanamycin resistance. Candidate mutant clones were confirmed by PCR screening using primers T1 and T2 (Table 2). The wild-type and deleted alleles could be differentiated on the basis of the size of the amplicon by agarose gel electrophoresis. The mutant strain was designated △*aspA*::*kan*.

**Table 2:** Primers used in this study

Primer	Sequence 5′- 3’	RE
aspAU1	CGGGGTACCCCCTAATGCAGAAGTAATTAA	*Kpn*I
aspAU2	CCTGCAGGATGCGGCCGCGCATTTCGAGTGATGAACAAGT	*Pst*I/*Not*I
aspAD1	CGCGGCCGCATCCTGCAGGATAACTGTTAATTTAACCGCA	*Not*I/*Pst*I
aspAD2	CGCGAGCTCAGCGTGTAAGCAATATTTTAG	*Sac*I
Kan-F1	ATAAGAATGCGGCCGCTCAGTGGAACGAAAACTC	*Not*I
Kan-F2	TGCACCTGCAGGTTAGAAAAACTCATCGAGCATC	*Pst*I
aspA1	ATGACAGTAACAAGAAAAGAAGT	一
aspA2	TTATTTATTCAACTTCGCTTTATAG	一
T1	TTCGGCATTTAGCAAACTGACGACG	一
T2	GGTGTCACACTTCCGTGCGTTAGAG	一
CaspA1	CGCGGATCCTTTAATGATACAAGGGCTATGCTCA	*BamH* I
CaspA2	GGCGTCGACTTATTTATTCAACTTCGCTTTATAGG	*Sal* I
M1	GAAGAGTGCAGTTGGCTTGCG	一
M2	AAATCGCGAGGAATACTGACG	一
qaspA1	TTGTGGGGCGTATGTGATGG	一
qaspA2	ACTGGGTTGACTTTTGCTGGC	一
q16s rRNA1	TCACCGCAACATTCTGATTT	一
q16s rRNA2	CATACAGAGGGCAGCGAGA	一

Restriction endonuclease (RE) cleavage sites introduced into primers are underlined

For complementation of *aspA* mutants strains in *P. multocida*, the amplification of the promoter sequence (328 bp) and coding sequence (1419 bp) of *aspA* gene using two primers, CaspA1/2 (Table 2), was cloned into pAL99S [40] to obtain the plasmid pAL99S-aspA. Then, the recombinant plasmid and an empty vector were transformed into the *aspA* mutant strain via electroporation respectively. The two strains were selected on TSA containing kanamycin and spectinomycin and further confirmed by PCR and RT-PCR using primers M1/2 (outside the multiple cloning site of pAL99S) and qaspA1/2 (Table 2). The complementary strain was designated C△*aspA*::*kan* and the control strain was designated △*aspA*::*kan* (pAL99S).

### Growth in complex media under aerobic conditions

We compared the growth rates among C48–1, △*aspA*::*kan*, C△*aspA*::*kan* and △*aspA*::*kan* (pAL99S) by determining the growth curves of the three strains in different media. Overnight cultures in TSB were centrifuged at 2300 g for 5 min and diluted to optical density OD_600_ = 1 before subculture at 1:100 in 5 ml TSB, LB, and MH media, respectively, and incubation at 37 °C with constant shaking under aerobic conditions. Samples were collected every hour for 18 h to determine the OD_600_ and create a growth curve.

We further explored the amino acid catabolism function of *aspA* by comparing the growth abilities of the parent and mutant strains in LB and MH in the presence or absence of fumarate. Overnight cultures in TSB were centrifuged and diluted to OD_600_ = 1 before subculture at 1:100 in 5 ml LB and MH media in the presence or absence of 20 mM fumarate (Sigma), respectively, followed by incubation at 37 °C with constant shaking under aerobic conditions. Samples were collected every hour for 18 h to determine the OD_600_ and create a growth curve. All growth experiments were performed twice independently with three replicates.

### Growth under anaerobic conditions

*AspA* was shown to be essential for growth under anaerobic conditions in *A. pleuropneumoniae* [5] and *C. jejuni* [17]. To determine if *aspA* was also related to anaerobic tolerance in *P. multocida*, we compared the abilities of C48–1, △*aspA*::*kan*, C△*aspA*::*kan* and △*aspA*::*kan* (pAL99S) to grow in TSB under anaerobic conditions. Briefly, overnight cultures in TSB were centrifuged and diluted to OD_600_ = 1 before subculture at 1:100 into 5 ml TSB medium under aerobic or anaerobic conditions, in a MACS-MG-1000-controlled atmosphere workstation (DW Scientific, Japan), and then incubated at 37 °C with constant shaking. The absorbance at OD_600_ was measured after 16 h. All growth experiments were performed twice independently with three replicates.

Moreover, we explored the growth abilities of the parent and mutant strains in TSB under aerobic or anaerobic conditions in the presence or absence of fumarate. Overnight cultures were centrifuged and diluted to OD_600_ = 1 before subculture at 1:100 in 5 ml TSB media under aerobic or anaerobic conditions in the presence or absence of 20 mM fumarate, respectively, followed by incubation at 37 °C with constant shaking. The absorbance at OD_600_ was measured after 16 h. All growth experiments were performed twice independently with three replicates.

### Growth under acid conditions

Overnight cultures in commercial TSB medium (pH = 7.3) were centrifuged at 2300 g for 5 min and diluted to OD_600_ = 1. Then the diluted cultures were subcultured at 1:100 in 5 ml TSB, pH = 7.3 or TSB, pH = 5.0 and then incubated at 37 °C with constant shaking for 1 h under aerobic conditions. The number of residual bacteria was counted by spreading serial dilutions onto TSA. All experiments were performed twice independently with three replicates.

### DPD-sensitivity assays

We explored the effects of DPD concentrations on the growth of wild-type and mutant *P. multocida*, respectively C48–1 and △*aspA*::*kan* by examining their iron-limited growth in TSB containing the iron chelator DPD at 0, 100, 150, 200, 250, and 300 μM. Briefly, overnight cultures in TSB were centrifuged at 2300 g for 5 min and diluted to OD_600_ = 1 before subculture at 1:100 into ml TSB medium containing 0, 100, 150, 200, 250, and 300 μM DPD, followed by incubation at 37 °C with constant shaking under aerobic conditions. The absorbance at OD_600_ was measured after 16 h. All growth experiments were performed twice independently with three replicates.

### Growth under iron-depleted conditions

We investigated the role of *aspA* in iron acquisition by comparing the ability of C48–1, △*aspA*::*kan*, C△*aspA*::*kan* and △*aspA*::*kan* (pAL99S) to grow in TSB in the presence or absence of DPD. Overnight cultures in TSB were centrifuged at 2300 g for 5 min and diluted to OD_600_ = 1 before subculture at 1:100 in 5 ml TSB medium containing 0 or 150 μM DPD, followed by incubation at 37 °C with constant shaking under aerobic conditions. Samples were collected every hour for 18 h to measure the OD_600_ and create a growth curve. All growth experiments were performed twice independently with three replicates.

### Iron utilization under iron-depleted conditions

We explored the effects of different iron ions, FeCl_3_ and FeSO_4_ (Takara), on the growth of *P. multocida* C48–1 and △*aspA*::*kan*, C△*aspA*::*kan* and △*aspA*::*kan* (pAL99S) under iron-depleted conditions. FeCl_3_ or FeSO_4_ was added to TSB at a final concentration of 100 μM, followed by overnight culture, centrifugation, and dilution to OD_600_ = 1 before subculture at 1:100 in 5 ml TSB containing 0 or 150 μM DPD and incubation at 37 °C, with constant shaking in presence of oxygen. The absorbance at OD_600_ was measured after 16 h. All growth experiments were performed twice independently with three replicates.

### Transcription of *aspA* under iron-depleted conditions

C48–1, △*aspA*::*kan* and C△*aspA*::*kan* were grown in TSB at 37 °C for 16 h with agitation. Overnight cultures in TSB were centrifuged at 2300 g for 5 min and diluted to OD_600_ = 1 before subculture at 1:100 in 5 ml TSB medium containing 0, 100, 150, and 200 μM DPD, followed by incubation at 37 °C with constant shaking in presence of oxygen to reach OD_600_ = 0.6. The strains were collected by centrifugation and total RNA were extracted using an RNeasy Mini Kit (Qiagen, Hilden, Germany). All RNA isolated from samples was DNase treated with DNA-Free (Sigma) and reverse transcribed into cDNA using an iScript cDNA Synthesis Kit (Promega, Wisconsin, USA) and oligo (dT) primers before quantitative PCR (qPCR). The mRNA expression levels of *aspA* and the reference gene *16 s rRNA* were quantified by real-time PCR using SYBR Green Master Mix (Roche Diagnostics, Shanghai, China). The primers used for real-time PCR were designed using Primer Premier 5 (Premier Biosoft, Palo Alto, CA, USA) (Table 2). Expression levels were normalized to *16 s rRNA* and presented as fold-change compared with the respective controls. All experiments were performed twice independently with three replicates.

### Biofilm-formation assay

Biofilm formation by C48–1, △*aspA*::*kan* and C△*aspA*::*kan* was assessed by crystal violet staining in 6-well plates (Sigma) as described previously [41]. Briefly, overnight cultures in TSB were centrifuged and diluted to contain approximately 10^6^ colony-forming units (CFU)/ml, and 200 μl of the standardized inoculum was inoculated in TSB with or without 150 μM DPD into triplicate wells in six-well plates. Uninoculated medium was used as a control. After incubation at 37 °C for 48 h under aerobic conditions, the final culture density was determined by measuring the OD_600_. The cells were then stained with crystal violet, washed, and the crystal violet was solubilized with 95% ethanol. The total biomass was quantified by measuring the OD_600_ of the dissolved crystal violet. To avoid any effects of different growth rates caused by DPD, the biomass of the biofilms was measured by crystal violet staining and expressed relative to the final culture density. All growth experiments were performed twice independently with three replicates.

### Assessment of virulence in vivo

We investigated the role of *aspA* in virulence in chickens in vivo. Briefly, healthy, 55-day-old healthy chickens were purchased from a chicken farm (HuBei, PR China) and housed in cages under a 12 h light/dark cycle. Throughout the experiment, chickens were kept at 25–28 °C and provided with food and water ad libitum. C48–1 and *aspA* mutant were grown in TSB at 37 °C for 16 h in TSB with agitation under aerobic conditions. Overnight cultures were then centrifuged and diluted to contain approximately 10 and 100 CFU, respectively. The chickens were divided randomly into five groups of 10 each and injected intramuscularly with 10 or 100 CFU of the wildtype C48–1 or mutant strain △*aspA*::*kan*. The negative control group was injected with phosphate-buffered saline (PBS). Chicken mortality was recorded daily for a period of 12 days after the challenge. The remaining chickens were killed humanely with an intravenous injection of sodium pentobarbital (100 mg/kg bodyweight) at the end of the study.

### In vivo competition assay

C48–1, *aspA* mutant and complemented strains were grown in TSB at 37 °C for 16 h with agitation. Overnight cultures were then centrifuged and diluted to contain approximately 10^3^ CFU/ml, respectively. C48–1 were then mixed with △*aspA*::*kan* or C△*aspA*::*kan* at a 1:1 ratio and 55-day-old healthy chickens were injected intramuscularly with 200 μl of the mixture containing 100 CFU of each strain. At 24 h and 72 h after infection, tissue samples (0.25 g) from spleens and livers of the chickens (five per group) were collected, weighed, triturated in 900 μl of PBS, and homogenized. Subsequently, the homogenates were 10-fold serially diluted, and 100 μl of the diluted suspensions were plated on TSA agar with or without kanamycin (100 μg/ml). The mutant (or complement) titer was calculated from the CFU recovered on TSA agar containing kanamycin, and the bacterial load of wild-type was obtained from the CFU recovered on TSA agar and subtracted the number of the mutant. The results were shown as the log10 competitive index.

### Statistical analysis

All statistical analyses were performed using GraphPad Prism 5 (GraphPad Software Inc., San Diego, CA, USA) software for Windows. Differences were evaluated using Student’s *t*-tests. The value of *P* < 0.05 was considered significant.

## Data Availability

The datasets generated and analysed during the current study are available from the corresponding author on reasonable request.

## Abbreviations

*AspA*: Aspartate ammonia-lyase
*P. multocida*: *Pasteurella multocida*
LB: Luria- Bertani
MH: Mueller-Hinton
*C. jejuni*: *Campylobacter jejuni*
*A. pleuropneumoniae*: *Actinobacillus pleuropneumoniae*
TSB: Tryptone Soya Broth
TSA: Tryptic Soy Agar
Kan^R^: Kanamycin-resistance
CFU: Colony Forming Unit
DPD: 2,2′-dipyridyl
